# Using Knowledge-Guided Machine Learning To Assess Patterns of Areal Change in Waterbodies across the Contiguous United States

**DOI:** 10.1021/acs.est.3c05784

**Published:** 2024-03-06

**Authors:** Heather L. Wander, Mary Jade Farruggia, Sofia La Fuente, Maartje C. Korver, Rosaura J. Chapina, Jenna Robinson, Abdou Bah, Elias Munthali, Rahul Ghosh, Jemma Stachelek, Ankush Khandelwal, Paul C. Hanson, Kathleen C. Weathers

**Affiliations:** † Virginia Tech, Blacksburg, Virginia 24060, United States; ‡ University of California, Davis, Davis, California 95616, United States; § Dundalk Institute of Technology, Dundalk A91 K584, Ireland; ∥ McGill University, Montréal, Quebec H3A 0B9, Canada; ⊥ University of Vermont, Burlington, Vermont 05401, United States; # Rensselaer Polytechnic Institute, Troy, New York 12180, United States; ∇ City University of New York, New York, New York 10031, United States; ○ Northern Region Water Board, Bloemwater Street, Mzuzu 105206, Malawi; ◆ University of Minnesota, Minneapolis, Minnesota 55455, United States; ¶ Los Alamos National Laboratory, Los Alamos, New Mexico 15672, United States; & University of Wisconsin − Madison, Madison, Wisconsin 53706, United States; ● Cary Institute of Ecosystem Studies, Millbrook, New York 12545, United States

**Keywords:** domain knowledge, KGML, K-means clustering, limnology, machine learning, surface area, temporal change

## Abstract

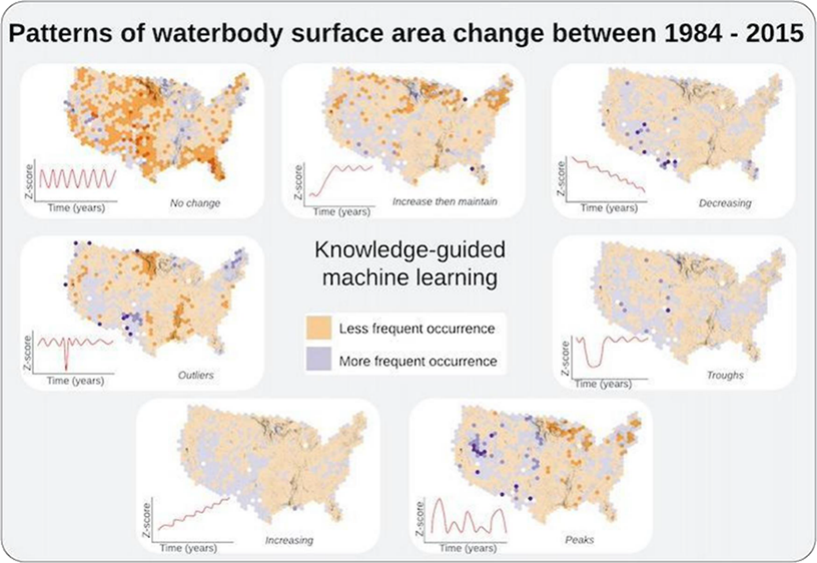

Lake and reservoir surface areas are an important proxy for freshwater availability. Advancements in machine learning (ML) techniques and increased accessibility of remote sensing data products have enabled the analysis of waterbody surface area dynamics on broad spatial scales. However, interpreting the ML results remains a challenge. While ML provides important tools for identifying patterns, the resultant models do not include mechanisms. Thus, the “black-box” nature of ML techniques often lacks ecological meaning. Using ML, we characterized temporal patterns in lake and reservoir surface area change from 1984 to 2016 for 103,930 waterbodies in the contiguous United States. We then employed knowledge-guided machine learning (KGML) to classify all waterbodies into seven ecologically interpretable groups representing distinct patterns of surface area change over time. Many waterbodies were classified as having “no change” (43%), whereas the remaining 57% of waterbodies fell into other groups representing both linear and nonlinear patterns. This analysis demonstrates the potential of KGML not only for identifying ecologically relevant patterns of change across time but also for unraveling complex processes that underpin those changes.

## Abstract 2

Knowledge-guided machine learning was used to classify ecologically interpretable long-term patterns in the surface area of 103,930 US waterbodies.

## 1 Introduction

The surface area of a lake or reservoir (hereafter both referred to as a “waterbody”) is an important indicator of freshwater availability and has been recognized as an “Essential Climate Variable” by the Global Climate Observation System.^1^ Waterbody surface areas oscillate naturally due to seasonal precipitation and evaporation patterns, and also as a result of anthropogenic stressors including societal water use, human land management, and climate change, which threaten waterbodies and the ecosystem services that they provide.^2^ These stressors alter water availability, physical properties (e.g., temperature regime, light penetration), and the composition, biomass, and biodiversity of ecological communities.^3−9^ For example, several waterbodies across the globe have experienced surface area declines, including large lakes in Central Asia (e.g., Aral Sea), Central Africa (e.g., Lake Chad), the Altiplano region (e.g., Lake Poopó), the Middle East (e.g., Lake Urmia), and the Western United States (e.g., Lake Mead and the Great Salt Lake). These changes have intensified water scarcity and affected commerce, access to drinking water, public health, and hydropower generation.^10−13^ In addition, regional lake area declines in the Mongolian Plateau have threatened the livelihood of local people,^14^ while waterbody expansions in North Dakota (USA) have displaced agricultural croplands and existing wetland vegetation.^15^ Lake expansions due to increasing precipitation, glacier melt, and permafrost thaw at high elevations (e.g., in the Tibetan Plateau,^16^ the Alps,^17^ and Patagonia^18^) serve as hydrological responses to global warming while increasing the risk for catastrophic glacial-lake outburst floods.

On regional to global spatial scales, long-term waterbody surface area changes have predominantly been assessed using statistical approaches, i.e., by quantifying linear trends^16,19−22^ and/or statistical variability.^23−26^ However, patterns of waterbody surface area change are not always linear and likely exhibit abrupt shifts, inconsistent oscillations, or other patterns of variability that reflect regional water balance fluctuations or human intervention.^19,26^ Increasing variability in lake ecosystems can be indicative of global change^27,28^ and is likely to affect their resilience to the negative effects of climate change.^29^ Characterizing how and where lakes are changing on broad scales is, therefore, critical to understanding the drivers of change. However, studies that have investigated patterns of waterbody surface area change, rather than quantifying variability or linear change, are typically performed on short-term time series (∼2 years),^23^ long-term but low-resolution time series (i.e., only five measurements in 22 years),^30^ or on few (<30) waterbodies.^31−33^ Recently, the Reservoir and Lake Surface Area Time series (ReaLSAT) data set, a long-term, spatially extensive water body data set, has become available^34^ which makes possible, using new analytic tools, a comprehensive analysis of long-term patterns of change across broad spatial scales.

Machine Learning (ML) has been used in the environmental sciences as a tool for analyzing time series patterns in large data sets using pattern recognition algorithms.^35^ To further interpret patterns, commonalities in patterns can be classified by using clustering algorithms. Such discretization of, for example, waterbodies, can facilitate ecological understanding, and ultimately management of these ecosystems.^36^ However, ML approaches identify patterns but do not include mechanisms as a basis for the interpretability of results.^37^ These patterns can therefore identify spurious relationships in training data sets that may not be indicative of ecological phenomena, making it difficult to extrapolate to out-of-sample data sets.^38^ To address these limitations, researchers have begun to expand the utility of ML by engaging scientific knowledge to guide ML algorithms. These knowledge-guided machine learning (KGML) approaches constrain typical ML models with domain knowledge of environmental science and have been shown to improve the performance of ML models while providing for interpretation of results in ways that are relevant to ecological phenomena.^39−42^

The introduction of KGML techniques in combination with the recent increased accessibility of high-quality remote sensing data products provides an opportunity to analyze and classify patterns of waterbody surface area change on broad spatial scales. The ReaLSAT data set^34^ is specifically suited for this purpose, as it provides 32 years of monthly predictions (January 1984–January 2016) of surface area for waterbodies (>0.1 km^2^) around the world. In addition, ReaLSAT provides dynamic waterbody polygons with unique lake identifiers, rather than static polygons^43,44^ or pixel-based surface water area time series that are not linked to specific waterbodies.^20,21,45^

In this study, we combine the ReaLSAT data set with KGML for the first time, to analyze and classify patterns in the surface area of 103,930 waterbodies across the contiguous United States. We employed KGML using a team science approach, combining domain knowledge from ecology, hydrology, and computer sciences.^46−48^ Our objectives were to (1) analyze and classify the long-term patterns of change in waterbody surface areas and (2) determine how those patterns differ across the contiguous United States. In addressing these objectives, we demonstrate how KGML and an interdisciplinary team science approach work in concert to effectively inform the design of the analytical framework and guide the interpretation of the results.

## 2 Methods

### 2.1 Study Area and Data Set Description

This study analyzed the patterns of change of 103,930 waterbodies of the contiguous United States using the ReaLSAT data set.^34^ This region was selected due to its high waterbody abundance and the large east–west gradient in climate, geology, and morphology, introducing a diversity of waterbody types that makes this region ideal for the purpose of this study.

The ReaLSAT data set used pixel-based land/water classification maps of the Global Surface Water data set (GSW) to create dynamic polygons for 681,137 lakes and reservoirs globally from 1984 to 2016 using a physics-guided machine learning algorithm.^34^ To correctly identify a waterbody, this algorithm required at least 100 GSW pixels (at a resolution of 0.9 arc-seconds or 30 m at the equator), therefore, waterbodies <0.1 km^2^ were not included. Nevertheless, ReaLSAT identified relatively small waterbodies (i.e., with surface areas ∼0.1 km^2^), including ephemeral and agricultural ponds that do not appear in other data sets because they were either not classified as a “lake” or “reservoir” or were not recognized because they are highly dynamic in their extent and existence. In addition, ReaLSAT included reservoirs on rivers and oxbow lakes (∼5% of nonlakes) in the data set, while acknowledging that they could not always be distinguished from river segments. In this study, we adopted ReaLSAT’s definition of “lakes and reservoirs”, which referred to all lentic (nonflowing) waters, including dynamically operated ponds. Limitations to the data set include the prevalence of data gaps, which are larger and more abundant before the year 2000 and during the winter season of most northern lakes (i.e., during ice cover). However, these gaps were filled in using the machine learning algorithm, further described below.

### 2.2 KGML Pattern Recognition and Clustering Methods

**Figure 1:**
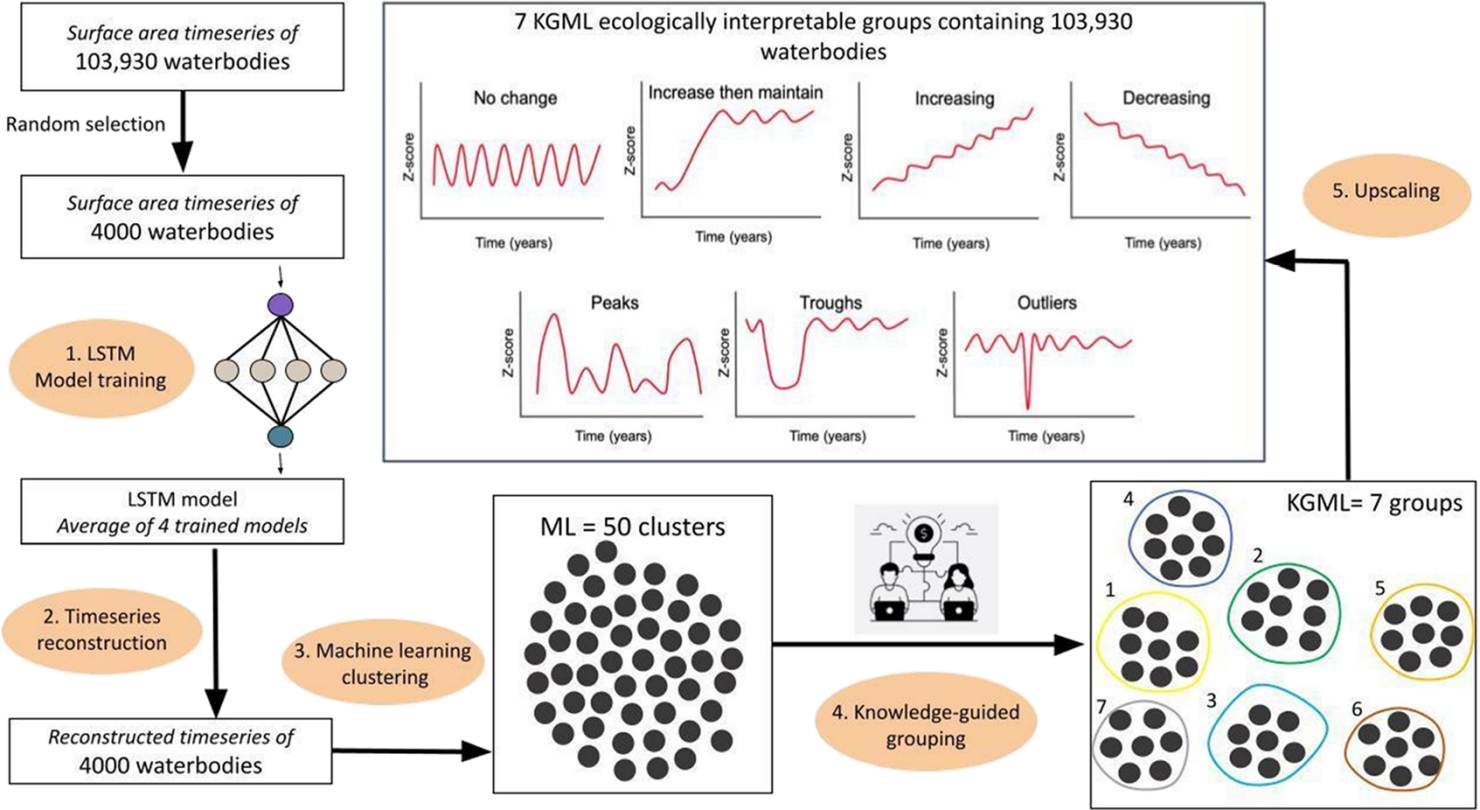
Methods for pattern recognition and clustering of surface area changes in 103,930 waterbodies of the contiguous United States from 1984–2016, using knowledge-guided machine learning (KGML). Black circles depict either ML clusters or KGML ecologically interpretable groups.

KGML is an approach that incorporates both domain knowledge of ecological phenomena and machine learning methods, allowing for ecological interpretability of results that is not necessarily feasible using unconstrained ML approaches.^37−39,41,42^ We used KGML to identify ecologically interpretable groups representing different patterns of waterbody surface area change over time in five steps (Figure 1): (1) we used a randomly selected subset of 4000 waterbodies (4% of the full data set) from the ReaLSAT time series to train several long short-term memory (LSTM) models (see Section 2.2.1 for model details). (2) The trained LSTM models with the lowest validation losses (four models) were averaged and used to produce smooth and gap-filled time series (i.e., “reconstructed” time series) for these 4000 waterbodies. (3) We then used machine learning (K-means clustering) to divide the low-dimensional embeddings for each of the 4000 waterbodies into 50 clusters sharing similar patterns of surface area change. (4) These 50 clusters were further combined into seven ecologically interpretable groups using domain knowledge (as described in Section 2.2.3) based on our physical understanding of waterbody area changes, which allowed us to distinguish ecologically plausible and interpretable patterns of surface area change. (5) Finally, we used K-means clustering and a statistical distance from the low-dimensional embeddings to the cluster centroids to group the remaining 99,930 waterbodies into one of the seven ecologically interpretable groups. The following sections describe these procedures in more detail.

#### 2.2.1 LSTM Model Training and Time Series Reconstruction

We trained a long short-term memory (LSTM) based sequence-to-sequence autoencoder model using the time series of 4000 randomly selected waterbodies (Figure 1, steps 1 and 2). We selected a small subset of waterbodies here to enable human experts to evaluate the results and provide ecological interpretability that was subsequently used to analyze the remaining waterbodies. All time series were z-score normalized to depict relative rather than absolute waterbody area changes. LSTM is a deep learning method that, when compared to more traditional neural networks, is particularly suited for the aims of our study because it captures long-term temporal dependencies^49,50^ necessary to identify waterbody dynamics. Furthermore, the autoencoder formulation provides a robust way to gap-fill missing time steps and remove outliers, addressing the issue of recurring data gaps in the ReaLSAT data set. This method returns a low-dimensional feature space that can be used for clustering. While there are some limitations to LSTM models, such as a greater amount of memory needed for training compared to other models,^51^ the advantages mentioned above made this model an excellent fit for our research objectives.

Because LSTMs are designed to run only forward in time and our objective was to maximize reconstruction performance irrespective of directionality, we used a bidirectional LSTM-based sequence encoder consisting of two LSTM structures: the forward LSTM and the backward LSTM. The two LSTM structures are similar, except that the time series is reversed for the backward LSTM (Text S1). The embeddings for the forward LSTM and backward LSTM were added to obtain the final embeddings. This representation was then fed through the LSTM decoder to produce a target sequence, which is the same as the input sequence in the encode-decode architecture. Specifically, we used a conditional decoder that iteratively extracted data at each time step based on the output data from the previous time steps. The autoencoder parameters were trained to maximize the likelihood of the data, which under the Gaussian assumption becomes the reconstruction loss computed as the mean-squared error between the reconstructed and the original time series (Figure S1). We selected the hyperparameters by iterative tuning (learning rate = 0.001, epochs = 1000, no. of clusters = 50, code dimensions = 64), resulting in average training and average validation losses of 0.37 and 0.39, respectively.

We averaged four trained LSTM models with the lowest training and validation losses to improve model performance.^52^ We used this ensemble of trained models to reconstruct the 4000-waterbody area time series. As described earlier, due to the autoencoder formulation, the reconstructed time series have no gaps or outliers.

#### 2.2.2 Machine Learning Clustering

Using the reconstructed time series from the trained LSTM model, we performed K-means clustering, an unsupervised machine learning algorithm, to group waterbodies into clusters based on their patterns of surface area change (Figure 1, step 3). We generated an elbow plot to identify the number of clusters necessary to capture most of the variation in our data set and determined that the optimal number was between five and ten (Figure S2). Then, we ran the K-means clustering algorithm for ten clusters, the maximum value identified by the elbow plot. We observed that some of these clusters depicted patterns of surface area changes that were ecologically similar. For instance, multiple clusters contained peaks in the surface area but were deemed separate because the timing of these peaks was different. We therefore determined that visual inspections and manual adjustments of the ML-derived clusters were necessary. We ran the K-means model again, but this time for 50 clusters, which we assumed to be the upper limit of ecologically possible and interpretable distinct waterbody types and which we could then use as a baseline for KGML grouping.

#### 2.2.3 Knowledge-Guided Grouping

We visually inspected the time series patterns of a random subset of waterbodies in each of the 50 clusters (Figure 1, step 4). In addition, we inspected satellite imagery using Google Earth and assessed the spatial distribution of all waterbodies in each cluster to assess whether each cluster represented similar waterbodies (i.e., from the same region, or ecological zone, or representing similar waterbody types). Through this analysis, we found that similar patterns of change were being divided into multiple clusters and that individual clusters did not clearly depict ecologically unique waterbodies. We then merged clusters with similar time series patterns into seven ecologically interpretable groups, with each of the seven groups representing a distinct pattern or type of surface area change over time. We used our domain knowledge of lake ecosystems to resolve slight differences in the merging process (e.g., whether an abrupt increase in the lake area was more important than its precise timing), during which we manually placed each of the 50 clusters into one of seven ecologically interpretable groups. Finally, we looked at the Euclidean distance (a measure of similarity, further described below) between each reconstructed time series and the centroid of each cluster of similar time series to confirm that the seven groups that we identified were reasonable for each waterbody (Figure S3).

#### 2.2.4 Scaling up to 103,930 Waterbodies

Once the randomly selected 4000 waterbodies were grouped into seven clusters, we used the trained LSTM model to create low-dimensional embeddings for the remaining 99,930 waterbodies (Figure 1, step 5). After pattern recognition, each waterbody was grouped into one of the 50 clusters based on the Euclidean distance between the waterbody’s reconstructed time series and the centroid of each of the 50 clusters, where the centroid of a cluster is defined as the multivariate mean of all waterbodies in that group. This resulted in 50 distances per waterbody, from which the smallest distance determined the group it was assigned to. The waterbodies were then sorted into the seven ecologically interpretable groups based on the criteria created on the subset of 4000 waterbodies (see Section 2.2.3). For instance, all waterbodies that were assigned to clusters 36 and 47 were grouped into the fifth ecologically interpretable group. We looked at the Euclidean distance between each reconstructed time series and the centroid of each cluster, across the seven groups, to confirm that there were no groups with obvious outliers (Figure S3). The group classifications for each waterbody can be linked via waterbody ID with the ReaLSAT data set and are available in the Zenodo repository.^53^

### 2.3 Comparing KGML versus ML Output

To compare KGML and ML approaches, we repeated step 3 (Figure 1, machine learning clustering) to generate seven clusters instead of 50. We then visually compared these seven clusters derived from machine learning to the seven ecologically interpretable groups produced using KGML (i.e., including step 4, the “knowledge-guided grouping”). The goal of this visual comparison was to determine how many of the clusters produced by using machine learning matched the ecologically interpretable groups produced by using KGML.

### 2.4 Spatial Analysis

To identify patterns in the spatial distribution of the clusters, we first binned the contiguous United States into 100 km^2^ hexagons. We then tested a null hypothesis, which assumes clusters were evenly spatially distributed, by using the binned hexagons. The null hypothesis (even spatial distribution) states that the percentage of waterbodies in each hexagon from a cluster (hex_%wb/clust_) is equal to the percent of waterbodies classified into that cluster from the entire data set of 103,930 waterbodies (total_%wb/clust_)
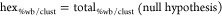
1

We tested the null hypothesis by subtracting total_%wb/clust_ from hex_%wb/clust_, resulting in either a positive or negative number (% difference)
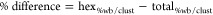
2

A positive number indicated that there were more waterbodies in the hexagon from a cluster than would be expected with an even spatial distribution. A negative number indicated that there were fewer waterbodies in the hexagon from that cluster than expected with an even spatial distribution. The resulting percent differences were z-score normalized to better compare the spatial distribution across clusters. All code and data files used to run these analyses are archived and available in the Zenodo repository.^53,54^

## 3 Results

### 3.1 Patterns of Change

We identified seven patterns of temporal surface area change from the 103,930 ReaLSAT waterbodies within the contiguous United States using KGML (Figure 1). Patterns were described using domain knowledge, as (1) no change over time, (2) substantial increase and then maintain, (3) steady increase over time, (4) steady decrease over time, (5) peaks, (6) troughs, and (7) outliers or patterns for which there was no apparent ecological mechanism (Table 1). Many waterbodies (43% of the total) were classified as having no surface area change over time (group 1), while the remaining 57% of waterbodies fell into one of the other six ecologically interpretable groups (Table 1).

**Table 1:** Overview of the Seven Ecologically Interpretable Groups Generated via Knowledge-Guided Machine Learning (KGML), Which Describes the Temporal Patterns in the Surface Area Change between 1984 and 2016 for 103,930 Waterbodies across the Contiguous United States

group number	percentage of lakes	short description	long description
1	43%	no change over time	waterbodies have repeated surface area oscillations over time; increases and decreases are similar in magnitude relative to the baseline
2	13%	substantial increase and then maintain	waterbody surface area increases dramatically early in the time series before leveling off with smaller oscillations toward the end
3	7%	steady increase over time	waterbodies have a consistent upward trend in surface area throughout the entire time series
4	6%	steady decrease over time	waterbodies have a consistent downward trend in surface area throughout the entire time series
5	11%	peaks	waterbodies generally have a stable baseline below a z-score of 0, from where the surface area peaks 1 to 3 times in the time series
6	6%	troughs	waterbodies generally have a stable baseline with a z-score between 0 and 1, with one trough in the time series where the surface area temporarily decreases
7	14%	outliers	waterbody surface area dramatically drops at various points in the time series and the z-score is more than 3 standard deviations away from the mean

### 3.2 Comparison of KGML vs ML Patterns of Surface Area Change

**Figure 2:**
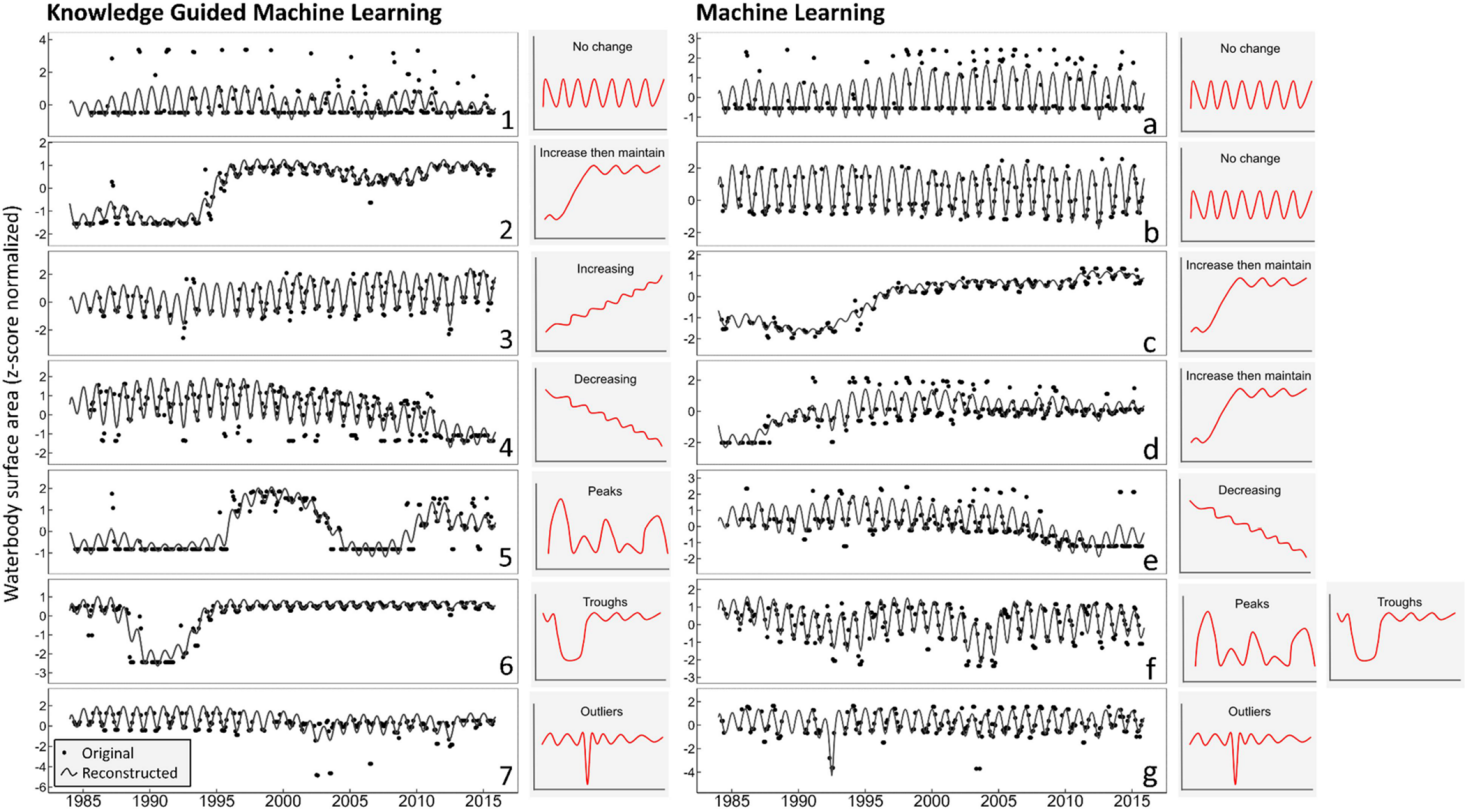
Waterbody surface area time series (1984–2016) in the contiguous United States for ecologically interpretable groups 1–7 that were derived by knowledge-guided machine learning (KGML) and clusters a–g that were derived by Machine Learning (ML). Each panel shows the original as well as reconstructed time series of a waterbody that is representative of its cluster, based on its proximity to the cluster centroid. Illustrations of the generalized KGML group patterns are indicated next to each panel, where the illustration associated with each ML panel indicates the ecologically interpretable group that KGML would have assigned each of the ML clusters.

Two of the seven groups produced via KGML, groups 4 (steady decrease over time) and 7 (outliers), were also identified as unique clusters using only ML (clusters e and g; Figure 2). The pattern of group 1 (no change over time) was detected by ML as well, however, based on differences in the scale of surface area fluctuations, ML subdivided the allocated time series into two separate clusters (a and b). Similarly, the pattern of group 2 (substantial increase then maintain) was detected by ML but allocated to two different clusters based on the timing and rate of the increase (c and d). One cluster identified by ML was not clearly defined using KGML (cluster f), as waterbodies that fell into this cluster either had patterns of “peaks” (group 5) or “troughs” (group 6). Finally, group 3 (a steady increase over time) was not identified by the ML method at all.

### 3.3 Spatial Distribution of KGML Ecologically Interpretable Groups

**Figure 3:**
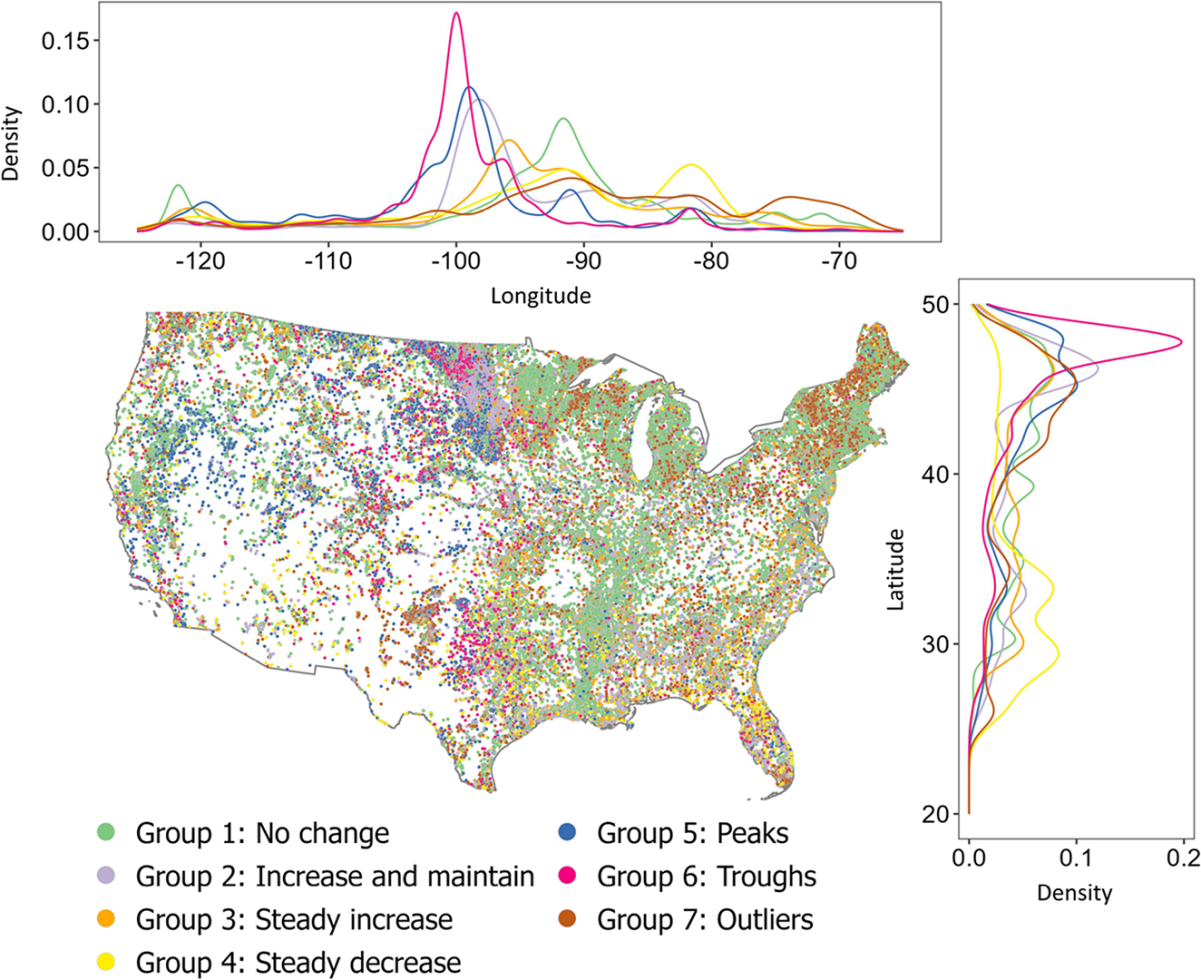
Map of 103,930 waterbodies within the contiguous United States. The associated temporal ecologically interpretable group classification of each waterbody is depicted by colored points on the map and colored lines in the side panels. Side panels represent the kernel density estimate of the latitudinal (right) and longitudinal (top) distributions of each waterbody group.

Different patterns in the spatial distribution of the seven ecologically interpretable groups were identified (Figure 3). Waterbodies in group 4 (steady decrease over time) were more abundant in the lower latitudes, whereas waterbodies in group 6 (troughs) were more abundant in the higher latitudes. Most groups had the highest densities in the midlongitude region, with small increases in density in western and eastern longitudes for groups 1 (no change over time) and 4 (steady decrease over time) (Figure S4).

**Figure 4:**
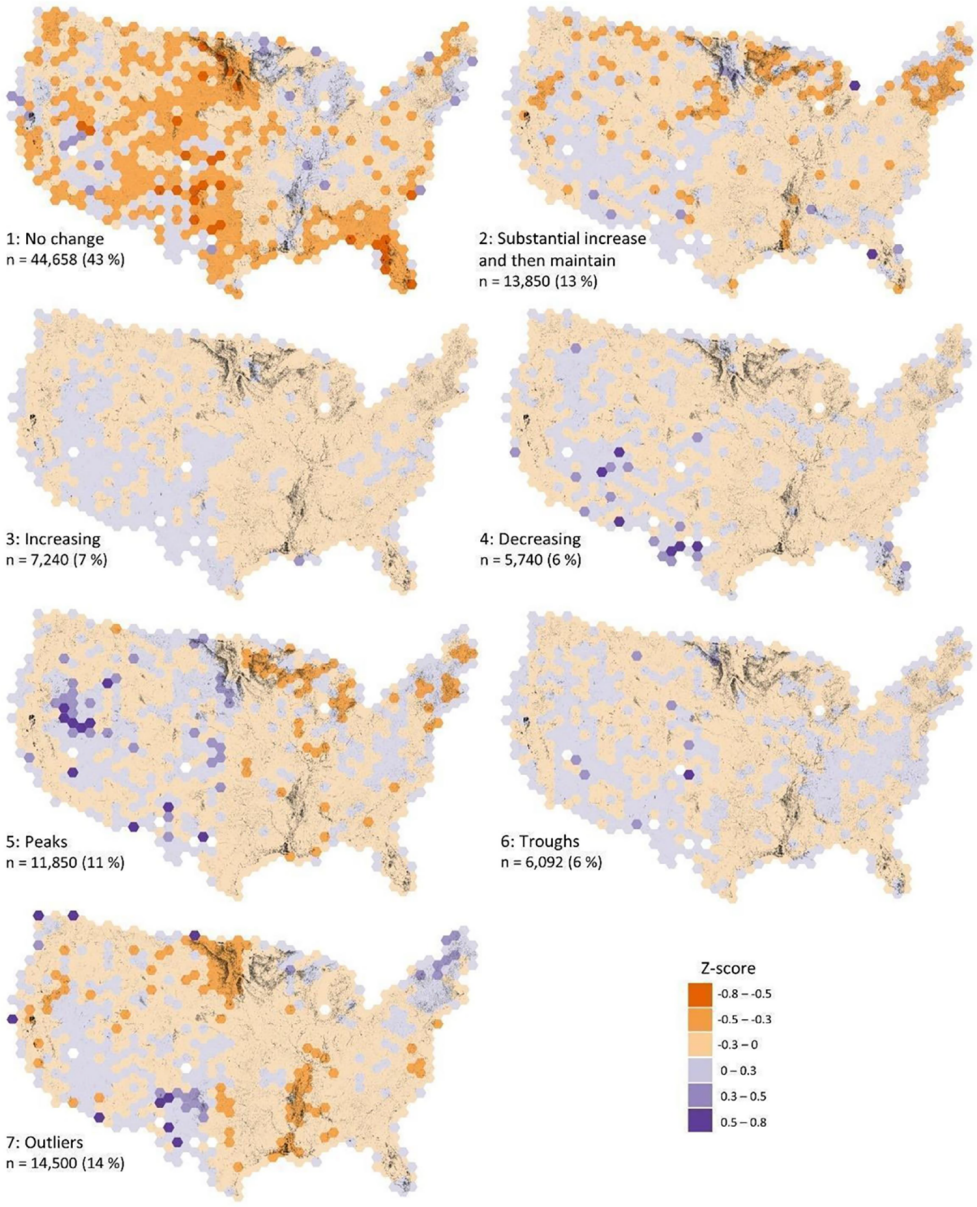
Spatial distribution of KGML ecologically interpretable groups for 103,930 waterbodies in the contiguous United States. Z-score values represent the normalized percent difference from the null hypothesis (even spatial distribution). Orange versus purple hexagons represent fewer versus more waterbodies than would be expected from an even distribution across the United States. Hexagons with no identified waterbodies are white. Spatial distribution within the contiguous United States, group description, number, and percentage of water bodies are depicted for each group.

We compared the spatial distribution of waterbodies in each of the seven groups to the null assumption that waterbodies from each cluster would be evenly distributed spatially across the full data set (103,930 waterbodies representing the contiguous United States; Figure 4). If a group had differences in spatial distribution beyond the null assumption, then there may be relationships between the waterbodies in each group and their geographic location. The occurrence of waterbodies with no change in surface area (group 1) was relatively high in most of the Mississippi River Valley and relatively low in most other regions of the contiguous United States. Additionally, there were more than average waterbodies with peaks and troughs in their surface area time series (groups 5 and 6) in western regions. Waterbodies exhibiting increases in surface area (groups 2 and 3) showed moderate deviations from the null assumption, with slightly higher abundance in the southwestern United States and localized high abundances in Florida and Michigan. Additionally, in the Southwest, there were more waterbodies with decreasing surface areas (group 4) but fewer in most other regions of the United States. Waterbodies with outliers (group 7) were more common in the Northeast and Southwest United States.

## 4 Discussion

### 4.1 Importance of a KGML and an Interdisciplinary, Team Science Approach

Addressing clear and concise science questions sometimes requires complex data, analytical resources, and an interdisciplinary team science approach.^46−48^ KGML instantiates both the technical and social underpinnings of those ideas, as it requires the integration of tools and knowledge from diverse domains.^37,39,40^ Our team consisted of ecologists, engineers, hydrologists, and computer scientists at different career stages who worked collaboratively and interactively throughout the entirety of this project, each learning and utilizing tools from others’ expertise. The emphasis on best practices of team science from idea development to interpretation and communication of results was an integral component of this work, as it allowed us to effectively leverage expertise from different skill sets within the group. Asking a question such as “What are the long-term patterns of change in U.S. waterbodies?” might appear simple, but it belies the challenges of extracting meaningful insights from a very large and complex data set using both ML tools and domain knowledge. The main findings of our work, identifying seven patterns of change and how those patterns are distributed across the U.S., reveal the importance of scale by showing that some decadal changes in waterbodies have regional-specific tendencies. Our findings also show the importance of the local scale, where neighboring waterbodies can exhibit strikingly different temporal patterns. While identifying the drivers of these patterns is the logical next step, fully addressing this question is beyond the scope of the present work. However, our results lay the foundation by providing a valuable data set and by showcasing the efficacy of team science in integrating a diverse set of human and technical resources to answer what initially seems like a straightforward inquiry.

The KGML approach explicitly incorporates domain knowledge into the analytical framework. We invoked expertise as part of the classification processes, resulting in groupings that differed in some cases from pure ML classification but were consistent with overall waterbody behavior. For example, we regrouped some of the clusters that ML allocated based on the timing of surface area changes and the scale of seasonal fluctuations. We observed that ML cluster c closely resembled ML cluster d, both matching KGML group 2 for waterbodies with surface area patterns that substantially increase and then maintain (Figure 2). The only difference between clusters c and d was the timing and intensity of the increase, while the overall patterns of surface area change were ecologically similar; i.e., they both likely represent a reservoir, filled at different times and rates after initial construction. We also found that ML did not capture surface areas that steadily increased over time, likely explained by the subtlety of this change compared to waterbodies experiencing no change over time or waterbodies with substantially increasing surface areas that then maintain. ML also produced a cluster that included multiple patterns identified using KGML, where the waterbodies had surface area patterns that resembled both peaks and troughs (groups 5 and 6), which could be an indication of either human management or heavy precipitation in different regions. By visually observing patterns in the ML reconstructed data, we identified waterbodies with steady increases in surface area over time, a subtle but important pattern compared to others with stronger signals. As a result, team science and KGML allowed us to effectively group waterbodies based on ecologically meaningful patterns of change.

### 4.2 Importance of Waterbody-Specific Data

A strong feature of our work, which is an extension of the approach used in the ReaLSAT data set, is a focus on individual waterbodies, rather than generalized pixel-based water surface area changes that can only address the more general notion of water on the landscape. Although pixel-based studies provide important information for the assessment of regional water storage, they do not tell us whether these changes in surface area are occurring across most waterbodies or are driven by relatively few large waterbodies. For example, Zou et al.^25^ and Pekel et al.^21^ determined that between 1984 and 2016, waterbodies in the western United States experienced strong surface area declines. Our analyses show that the relative number of waterbodies with decreasing surface area patterns was indeed above the national average in states like Utah and Nevada, but not in California, Oregon, or Washington (Figure 4). Knowing the ecologically interpretable group for each individual waterbody in these states could be useful for identifying the specific drivers that are causing different patterns of surface area change, which can then be used to implement water management strategies for specific waterbodies. Further, because patterns of surface area change are likely influenced by waterbody- and watershed-specific drivers, such as hydrology, morphology, climate, and anthropogenic factors, classifying waterbodies into ecologically interpretable groups is the first step toward fully understanding the mechanism driving these changes in surface area over time.

### 4.3 Potential Drivers of Long-Term Patterns of Waterbody Surface Area Change

Explaining waterbody surface area change is challenging due to the interaction of multiple climatological, hydrological, morphological, and anthropogenic variables across spatial and temporal scales. While identifying causal mechanisms for waterbody area change was beyond the scope of this study, we offer a few plausible interpretations meant to provide ecological context and process for our KGML model. In addition, we performed a preliminary driver analysis meant to inspire further investigation into waterbody area changes.

#### 4.3.1 Plausible Interpretations of Patterns of Surface Area Change

Group 1 can be ecologically defined as waterbodies that experience only minor oscillations in the surface area with no long-term or abrupt change. This can occur both naturally, such as in waterbodies located in regions with consistent hydrologic inputs, and in human-made systems, where water levels are manually controlled with infrastructure, such as pumps or dams. Group 2 can be ecologically explained as marking the creation of a waterbody, either by a large natural flooding event or by the intentional creation of a human-made reservoir. Groups 3 and 4 represent waterbodies in a long-term unidirectional trend, where they may experience a regular increase (group 3) or decrease (group 4) in surface area. For example, increased water inputs could be due to a steady increase in precipitation over multiple years, while decreasing water inputs could be due to long-term water extraction or drought. Groups 5 and 6 represent waterbodies that experience either a sudden increase (group 5) or decrease (group 6) in surface area over a short period (e.g., 1–5 years) but then return to a stable baseline. These periodic extreme water level fluctuations could reflect short-term anthropogenic changes in water use, such as temporary flooding and then draining of agricultural fields, or large water extractions from reservoirs. Short-term water fluctuations could also reflect natural phenomena, such as beaver damming, flash flood events, or isolated drought periods. Group 7 represents a waterbody surface area time series that experienced various extreme fluctuations over time. These are classified as “outlier” waterbodies that likely have case-specific explanations for their changes in surface area that cannot be generalized on this scale. In many cases, these ecologically uninterpretable fluctuations are caused by small data inconsistencies, which we observed via visual inspection of a subset of waterbodies within this group. Indeed, the ReaLSAT documentation describes potential sources of error causing false water or land detections, including the impact of surface algae and floating aquatic plants, the spurious water level fluctuations of agricultural ponds distorting the deep learning model used to fill in missing pixels, and missing data or low-confidence land-water classifications by the underlying Global Surface Water data set.^21^

#### 4.3.2 Preliminary Driver Analysis

Temporal change in the waterbody area, represented by the ecologically interpretable groups in our study, is unevenly distributed across the United States, suggesting that geographical drivers (latitude, longitude, and elevation) or climatic drivers (air temperature and precipitation) are important factors. A vast collection of potential drivers could be important. As a preliminary exploration, we focused on air temperature, precipitation, and elevation for an initial assessment because of their direct connection to the water cycle and because the data sets were readily available. We used gridded monthly air temperature and precipitation data from the National Oceanic and Atmospheric Administration Physical Sciences Lab in Boulder, Colorado, USA^55^ and elevation data from the United States Geological Survey^56^ to extract monthly time series for each waterbody (see Text S2), which we explored with a principal component analysis (Figures S5–9). We found that air temperature and precipitation likely both play a role in waterbodies with long-term decreasing trends in surface area (group 4), as these waterbodies were most prevalent in the hot climates of the southwest United States and Florida (Figure 4). These findings are in line with previous reports where air temperature was found to enhance evaporation and thus decrease waterbody surface area, particularly in the southern United States.^57^ In contrast, the northeast has waterbodies that exhibit substantial increases in surface area, especially along the Mississippi River, which might be best explained by the above-average precipitation of those regions, also reported by Zhang et al.^16^ Areas of high precipitation are associated with greater hydrologic connectivity in surface waters^58^ and lake-specific characteristics such as hydrologic connectivity are known drivers of lake surface area change.^59^ We also found that elevation best-explained waterbodies with peaks and troughs in their surface area (groups 5 and 6), which could occur if waterbodies at high elevations are exposed to greater precipitation and lower evaporation.^60^

Previous work on the spatial distribution of lake water quality^61,62^ has shown that, in many cases, there is no good explanation for some spatial patterns, and that in some cases, neighboring lakes that experience the same land use and climate can have very different water quality.^61^ It is possible that the same is true for waterbody area change over time as factors specific to a waterbody, such as its morphology, hydrology, or the existence of control structures, such as dams, override the influence of regional-scale drivers. Other factors are likely to explain surface area changes in waterbodies and will be important for future exploration. Examples include evaporation dynamics, known to be the main water loss for most waterbodies,^20,63^ hydrological connections, runoff, lake morphology,^64^ and anthropogenic influences (e.g., land-use change, urbanization, population density, agriculture, industry, mining).^65,66^ Ultimately, our KGML approach proved important for generating temporal patterns of waterbody change, whereby driving mechanisms can be explored. The resultant spatial distribution of surface area change across the contiguous United States was not random, suggesting that many of the long-term changes in the waterbody surface area are likely to have explanatory drivers, including broad-scale patterns in climate variables. Understanding patterns of change through the application of KGML is a crucial step in addressing the complex processes driving these changes, and we must understand both pattern and process to manage, protect, and build resilience for waterbodies and the critical ecosystem services they provide, especially in an era of profound change.

## Data Availability

All data^63^ and code^64^ necessary to recreate analysis in this manuscript are publicly available on Zenodo (https://zenodo.org/records/10207055; https://zenodo.org/records/10214420)

## Supporting Information Available

LSTM equations and architecture, Euclidean distances for KGML clusters, spatial distributions of clusters, elbow plot for determining the optimal number of clusters, alternate PCs to those shown in the main text, and results from the Tukey-HSD posthoc test (PDF)
