# Supplementary material for: Using Knowledge-Guided Machine Learning To Assess Patterns of Areal Change in Waterbodies across the Contiguous United States
Source: Environ Sci Technol. 2024 Mar 6;58(11):5003–13. doi: 10.1021/acs.est.3c05784 (PMC10956424; doi:10.1021/acs.est.3c05784)
Supplement: Supplementary file 1 — es3c05784_si_001.pdf [file es3c05784_si_001.pdf]

***finalSupplement to*: Using knowledge-guided machine learning to assess patterns of areal change in waterbodies across the contiguous U.S.**

Heather L. Wander[1*], Mary Jade Farruggia[2], Sofia La Fuente[3], Maartje C. Korver[4], Rosaura J. Chapina[5], Jenna Robinson[6], Abdou Bah[7], Elias Munthali[8], Rahul Ghosh[9], Jemma Stachelek[10], Ankush Khandelwal[9], Paul C. Hanson[11], Kathleen C. Weathers[12]

Submitted as an Article to *Environmental Science & Technology*

Number of Pages: 14
Number of Texts: 2
Number of Tables: 1
Number of Figures: 9
Number of References: 9

[1]Virginia Tech, Blacksburg, VA 24060, USA
[2]University of California, Davis, Davis, CA 95616, USA
[3]Dundalk Institute of Technology, Dundalk A91 K584, Ireland
[4]McGill University, Montréal, QC, H3A 0B9, Canada
[5]University of Vermont, Burlington, VT 05401, USA
[6]Rensselaer Polytechnic Institute, Troy, NY 12180, USA
[7]City University of New York, New York, NY 10031, USA
[8]Northern Region Water Board, Bloemwater Street, Mzuzu 105206, Malawi
[9]University of Minnesota, Minneapolis 55455, USA
[10]Los Alamos National Laboratory, Los Alamos, NM 15672, USA
[11]University of Wisconsin - Madison, Madison, WI 53706, USA
[12]Cary Institute of Ecosystem Studies, Millbrook, NY 12545, USA
[*]Email: hwander@vt.edu

Text S1; Long Short-term Memory model equations

Each LSTM (forward and backward) used the following equations to generate the embeddings for a sequence:

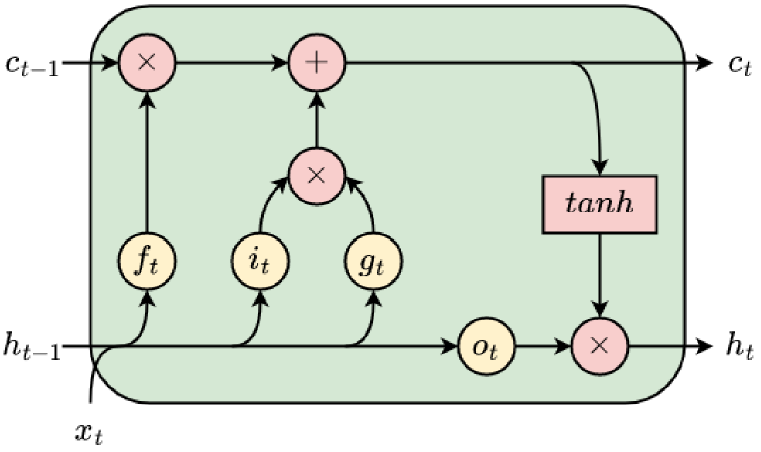

$$
\begin{aligned}
\boldsymbol{i}_t &= \sigma(\boldsymbol{W}_i \left[\boldsymbol{x}^t; \boldsymbol{h}^{t-1}\right] + \boldsymbol{b}_i) \\
\boldsymbol{f}_t &= \sigma(\boldsymbol{W}_f \left[\boldsymbol{x}^t; \boldsymbol{h}^{t-1}\right] + \boldsymbol{b}_f) \\
\boldsymbol{g}_t &= \sigma(\boldsymbol{W}_g \left[\boldsymbol{x}^t; \boldsymbol{h}^{t-1}\right] + \boldsymbol{b}_g) \\
\boldsymbol{o}_t &= \sigma(\boldsymbol{W}_o \left[\boldsymbol{x}^t; \boldsymbol{h}^{t-1}\right] + \boldsymbol{b}_o) \\
\boldsymbol{c}_t &= \boldsymbol{f}_t \odot \boldsymbol{c}_{t-1} + \boldsymbol{i} \odot \boldsymbol{g}_t \\
\boldsymbol{h}_t &= \boldsymbol{o}_t \odot \tanh\left(\boldsymbol{c}_t\right)
\end{aligned}
$$

Where, h_{t-1} and h_t are the hidden state at the previous time-step and current time step (short-term memory). c_t is the cell state at the current time-step (long-term memory). x_t is the input at the current time step. These are processed using i_t, f_t, g_t and o_t that are the input gate, forget gate, cell gate and output gate, respectively. The process equations are shown in the figure.

Text S2: Principal Component Analysis

Notably, as two similar methods of dimension reduction, PCA and K-means clustering are expected to give similar results and improved clustering accuracy has been observed when using both methods together.[1] Therefore, we performed a principal component analysis (PCA) to identify the environmental drivers that best explain the seven patterns of waterbody area change identified using KGML. For each waterbody, we used publicly available air temperature, precipitation, elevation,[2,3] latitude and longitude from the ReaLSAT dataset, and average surface area generated from the LSTM output. All driver data were z-score normalized before analysis. Using the PCA output, we then identified whether the centroids of each group were significantly different from each other with a PERMANOVA test (*adonis2()* function in the *vegan* R package),[4,5] followed by Tukey Honest Significant Difference (HSD) test to determine which of the seven centroids were significantly different from each other (*TukeyHSD()* function in the *stats* R package).[6] Given the size of the dataset, we randomly selected ~10% of waterbodies to determine whether group centroids differed from each other. All statistical analyses were conducted in R v.4.2.0. All code and data files used to run these analyses are archived and available in the Zenodo repository.[7,8]

We applied a PCA, using air temperature, precipitation, and elevation as potential explanatory drivers of the patterns in waterbody surface area change over time. Each of these climate variables can feasibly affect waterbody surface area via different mechanisms. For example, average precipitation directly influences surface area and air temperature is related to evaporation, which is a key component in explaining patterns of waterbody surface area change. Additionally, we included elevation, latitude, and longitude because differences among these variables can also affect surface area. For example, high elevation may be associated with high precipitation and lower mean air temperature,[9] high UV radiation,[10] or decreased anthropogenic influence,[11] which may alter patterns of waterbody surface area change. We used gridded monthly terrestrial air temperature and precipitation data from 1900-2014 provided by the NOAA PSL, Boulder, Colorado, USA (https://psl.noaa.gov) and the Global Multi-resolution Terrain Elevation Data provided by USGS (https://www.usgs.gov).[2,3] Because precipitation, air temperature, and elevation data had a spatial extent of 0.5° X 0.5°, we created polygons with the same spatial resolution to match waterbodies from the ReaLSAT dataset with their corresponding environmental data.

The scree plot indicated that the first two dimensions explained most of the total variance within the dataset (67%), therefore we focused further analysis on these two dimensions. (Figs. S5-S8). The seven waterbody types identified using KGML did not fall into distinct groups, as many of these groups were overlapping and no distinct spatial distribution was visible from the ordination (Fig. S5). We also ran a PERMANOVA[5] followed by a Tukey HSD test to identify significantly different means and found that waterbodies with a constant surface area over time and those whose surface area substantially increased before leveling off (groups 1 and 2) were significantly different from waterbodies with a steady increase in area over time, those with peaks, and those with troughs in surface area (groups 3, 5, and 6, $p < 0.05$; Table S1). Waterbodies with outliers in surface area trends over time (group 7) were significantly different from waterbodies with peaks or troughs in surface area (groups 5 and 6, $p < 0.05$, Table S1). Conversely, waterbodies that had constant surface area over time and those with surface areas that substantially increased before leveling off (groups 1 and 2) were similar to waterbodies with a steady decrease in surface area over time and those with outliers in surface area (groups 4 and 7; $p > 0.05$). Finally, waterbodies with peaks and troughs in surface area (groups 5 and 6) were also similar to waterbodies with a steady increase or decrease in surface area over time (groups 3 and 4, $p > 0.05$; Table S1).

Based on the PCA ordination, ecologically interpretable group centroids appeared to be better explained by different environmental drivers. Waterbodies that showed 1) no temporal change in surface area, 2) had a substantial increase and then maintained surface area and, 3) those that had a steady increase in surface area over time (groups 1-3) were better explained by mean precipitation and longitude, whereas waterbodies that had a steady decrease in surface area over time (group 4) were better explained by mean temperature (Fig. S5).

Table S1: Tukey HSD post-hoc test analyzing differences between each of the seven KGML group centroids, where groups represent waterbodies with the following surface area changes: 1) no change over time, 2) substantial increase and then maintain, 3) steady increase over time, 4) steady decrease over time, 5) peaks, 6) troughs, and 7) outliers or patterns that are not ecologically explained. Bold values indicate significance ($p < 0.05$).

Group	Difference	Lower 95% CI	Upper 95% CI	p-value
2-1	-0.0257	-0.151	0.099	0.997
3-1	0.127	0.002	0.253	**0.043**
4-1	0.065	-0.061	0.190	0.733
5-1	0.150	0.025	0.275	**0.008**
6-1	0.180	0.054	0.305	**0.000**
7-1	0.019	-0.106	0.144	0.999
3-2	0.153	0.028	0.278	**0.006**
4-2	0.090	-0.035	0.215	0.337
5-2	0.176	0.051	0.301	**0.001**
6-2	0.205	0.080	0.330	**0.000**
7-2	0.044	-0.081	0.169	0.943
4-3	-0.063	-0.188	0.062	0.755
5-3	0.022	-0.103	0.148	0.998
6-3	0.052	-0.073	0.177	0.883
7-3	-0.109	-0.234	0.016	0.137
5-4	0.085	-0.040	0.211	0.406
6-4	0.115	-0.010	0.240	0.095
7-4	-0.046	-0.171	0.793	0.934
6-5	0.030	-0.095	0.155	0.993
7-5	-0.131	-0.256	=0.006	**0.032**
7-6	-0.161	-0.286	-0.036	**0.003**

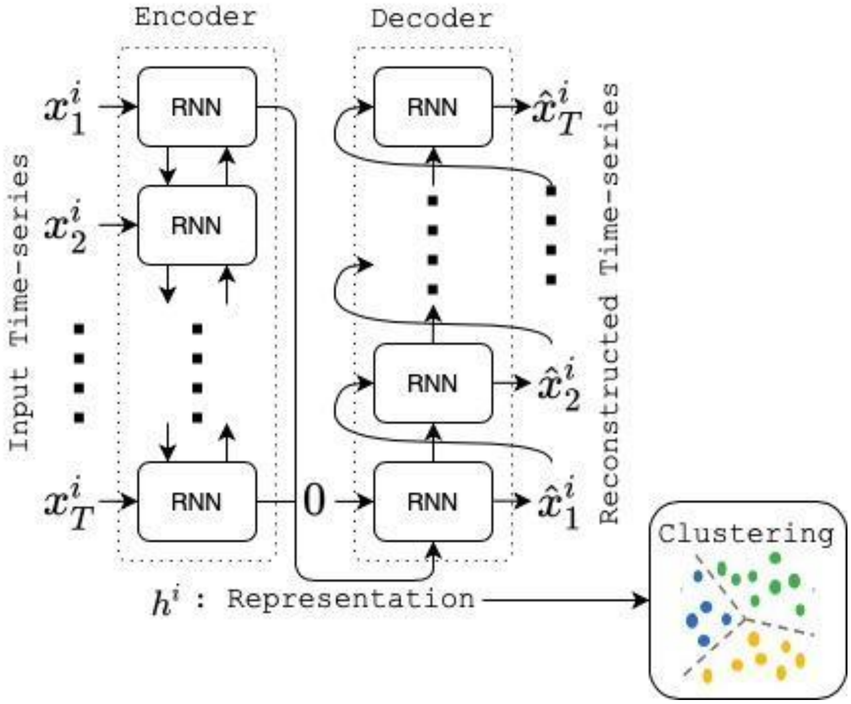

Figure S1. Recurrent neural network (RNN) used to reconstruct missing data from timeseries. This architecture consists of bi-directional Long Short-Term Memory (LSTM) encoder and single directional LSTM decoder. The encoder extracts a deep representation from the input time-series which is then fed into the decoder where the time-series is reconstructed. The representation extracted by the encoder is used for clustering the waterbodies using the K-means method.

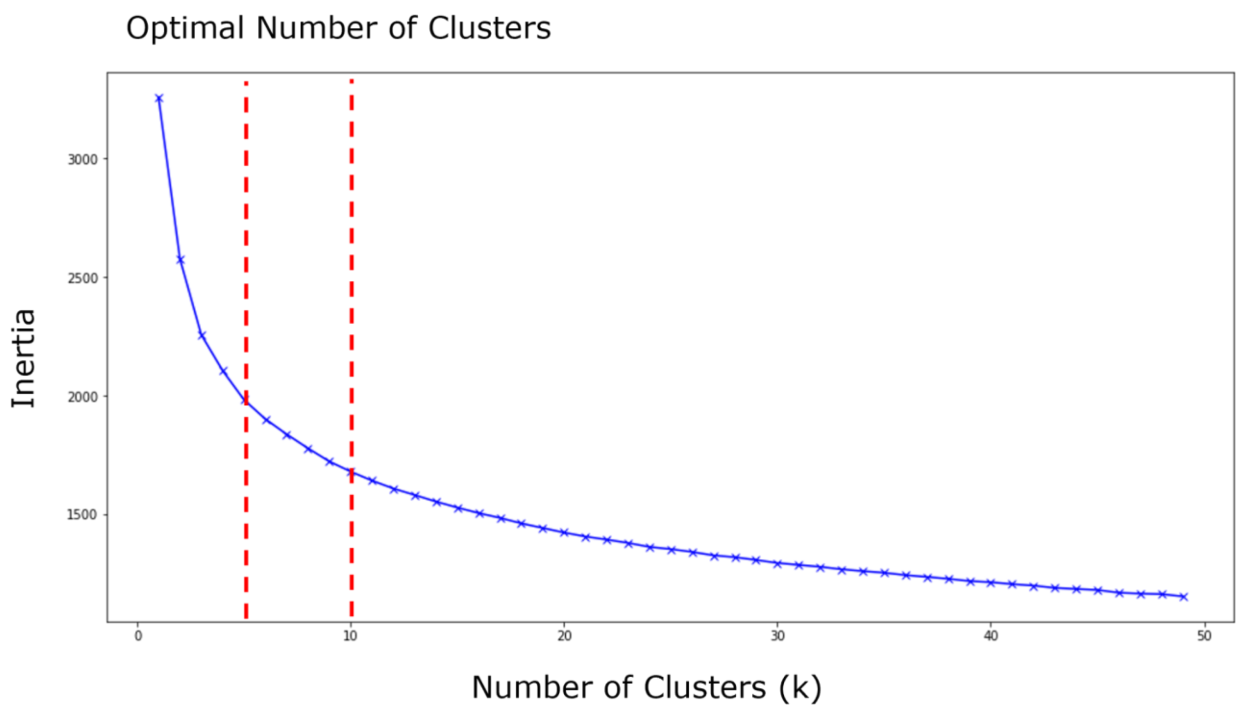

Figure S2. Elbow plot of K-means clustering of 400 waterbody timeseries. The elbow plot depicts the inertia (y-axis), or Sum of Squares Errors (SSE) from 1 to 50 clusters. The red lines depict the range of the optimal number of clusters that provide a low inertia but also minimize the number of clusters. An optimal number of seven clusters was chosen after visual inspection of cluster outputs based on domain knowledge of ecological patterns.

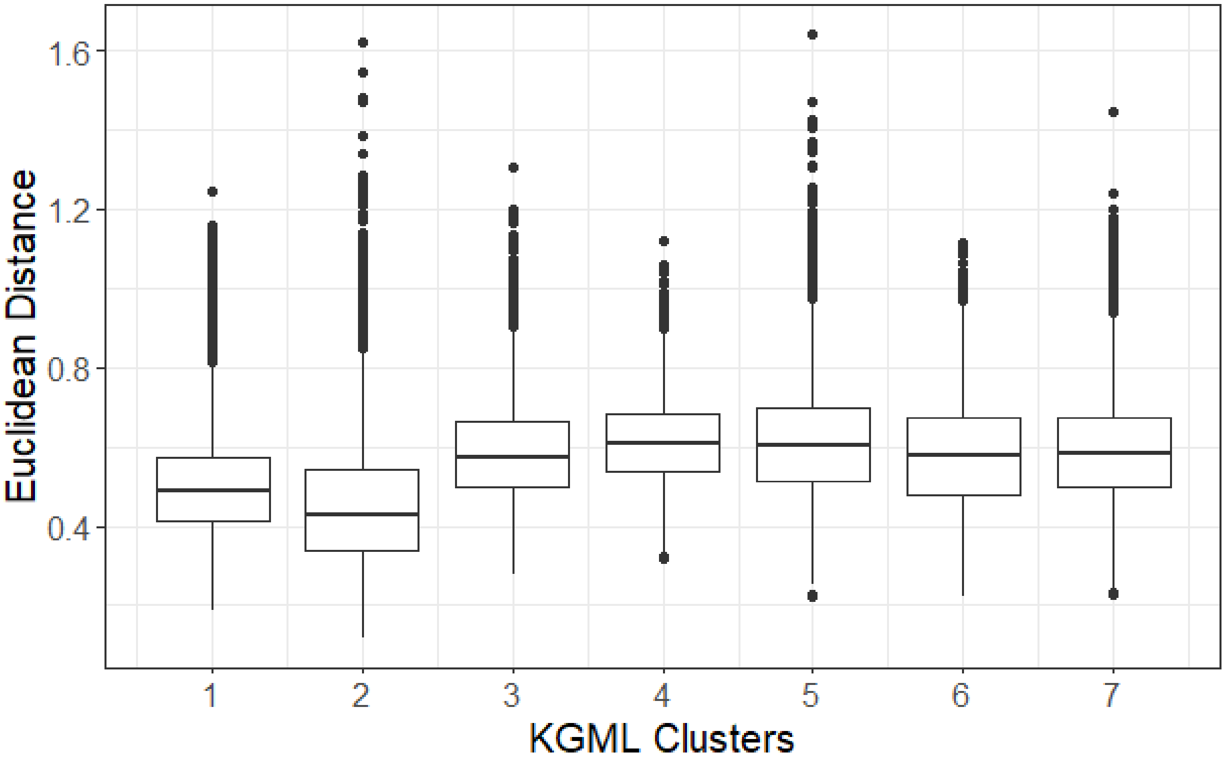

Figure S3. Boxplots showing Euclidean distance or similarity between each waterbody (n=103,930) and the centroid of each of the seven knowledge-guided machine learning (KGML) groups. Boxplots indicate the mean ± one quartile; the whiskers extend to the 5th and 95th percentile.

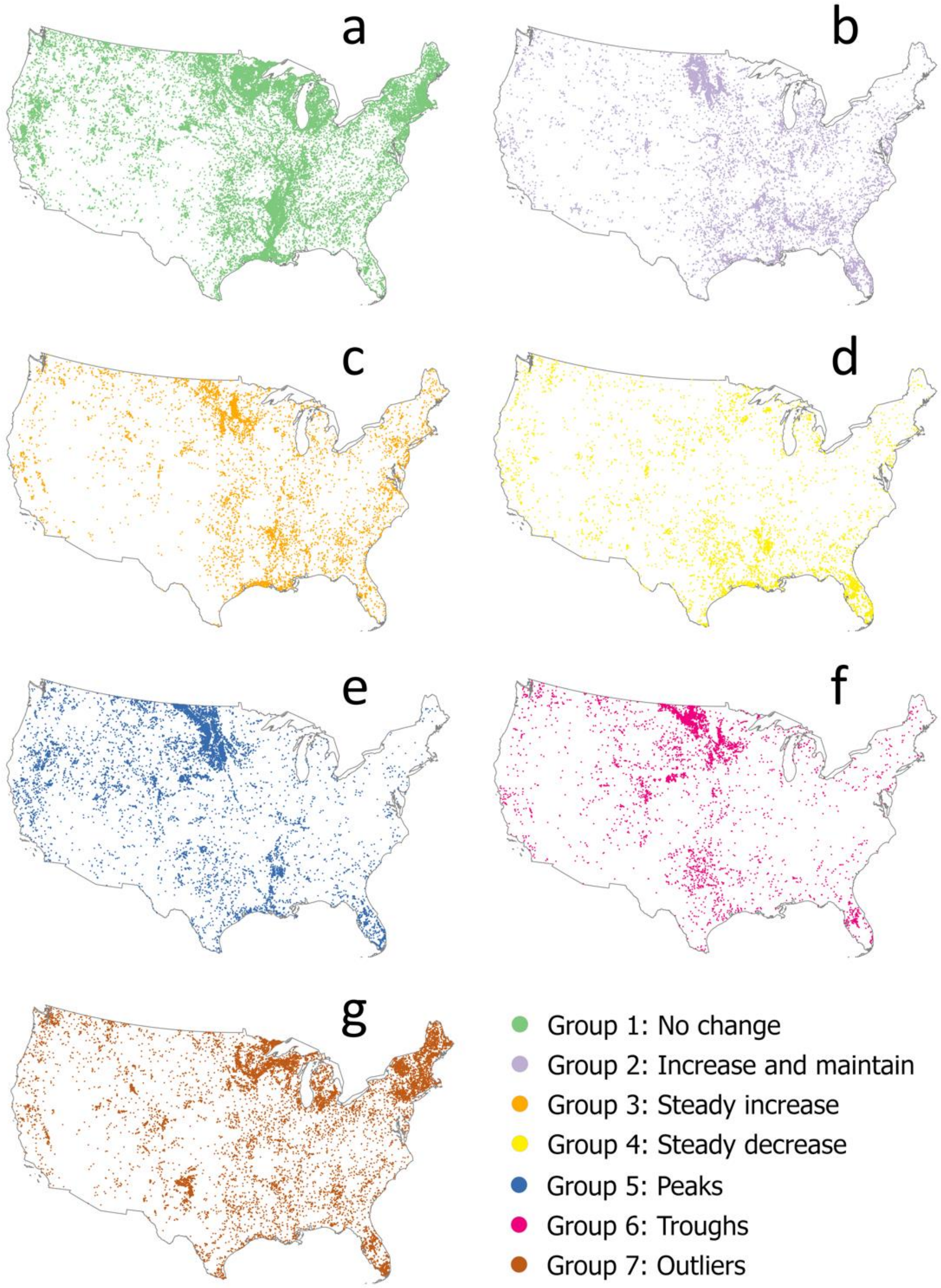

Figure S4. Spatial distribution of ecologically interpretable groups in the contiguous U.S. Shown are a) group 1, waterbodies with no change in SA; b) group 2, substantial increase in SA waterbodies and then maintain; c) group 3, waterbodies with steady SA increase over time; d) group 4, waterbodies with steady SA decrease over time; e) group 5, waterbodies with peaks; f) group 6, troughs; and g) group 7, waterbodies with presence of outliers.

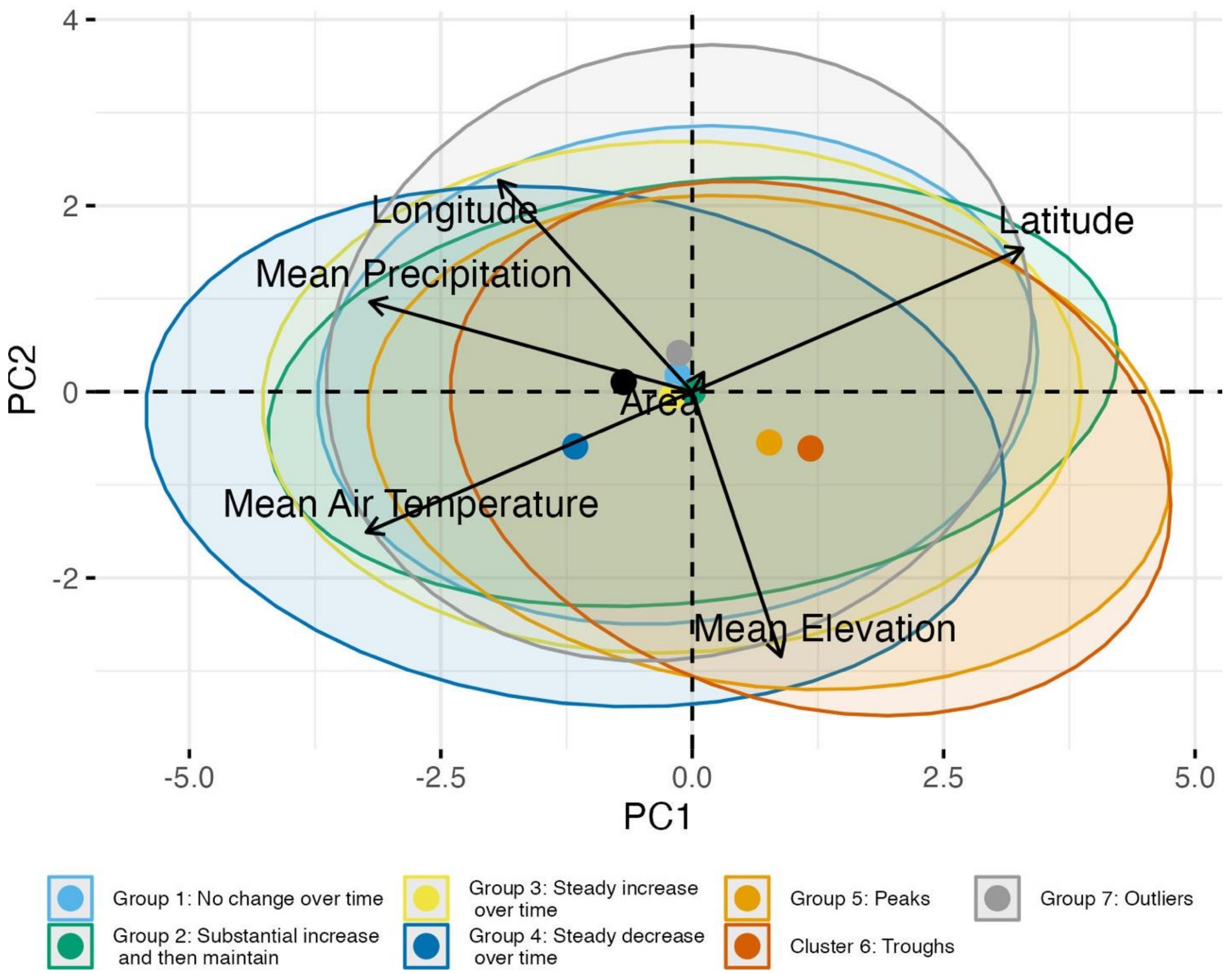

Figure S5. PCA biplot of all seven waterbody groups identified via knowledge-guided machine learning (KGML) with various environmental drivers. Eigenvectors depict correlations between driver data and each of the seven groups. The colored points represent the centroid of each groups and the black point represents the centroid of all 103,930 waterbodies across the contiguous United States. The first principal component (PC1) accounts for 44% of the total variance, whereas the second principal component (PC2) accounts for 23% of the total variance within the dataset, explaining a total of 67% of the variance.

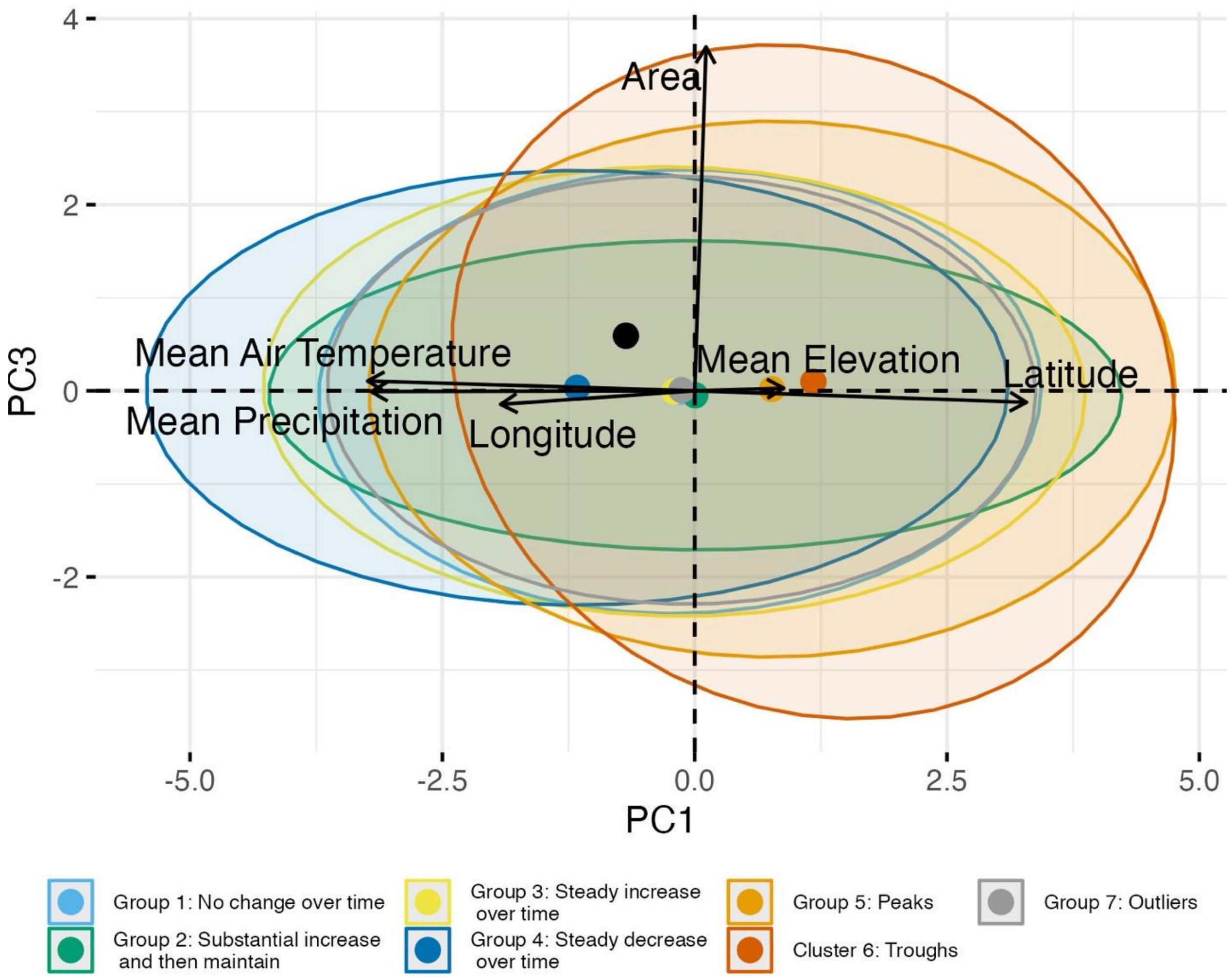

Figure S6. PCA biplot of all seven waterbody groups identified via knowledge-guided machine learning with various environmental drivers. Eigenvectors depict correlations between driver data and each of the seven groups. The colored points represent the centroid of each group and the black point represents the centroid of all 103,930 waterbodies. The principal component on the x-axis (PC1) accounts for 43.8% of the total variance, whereas the principal component on the y-axis (PC3) accounts for 16.6% of the total variance within the dataset.

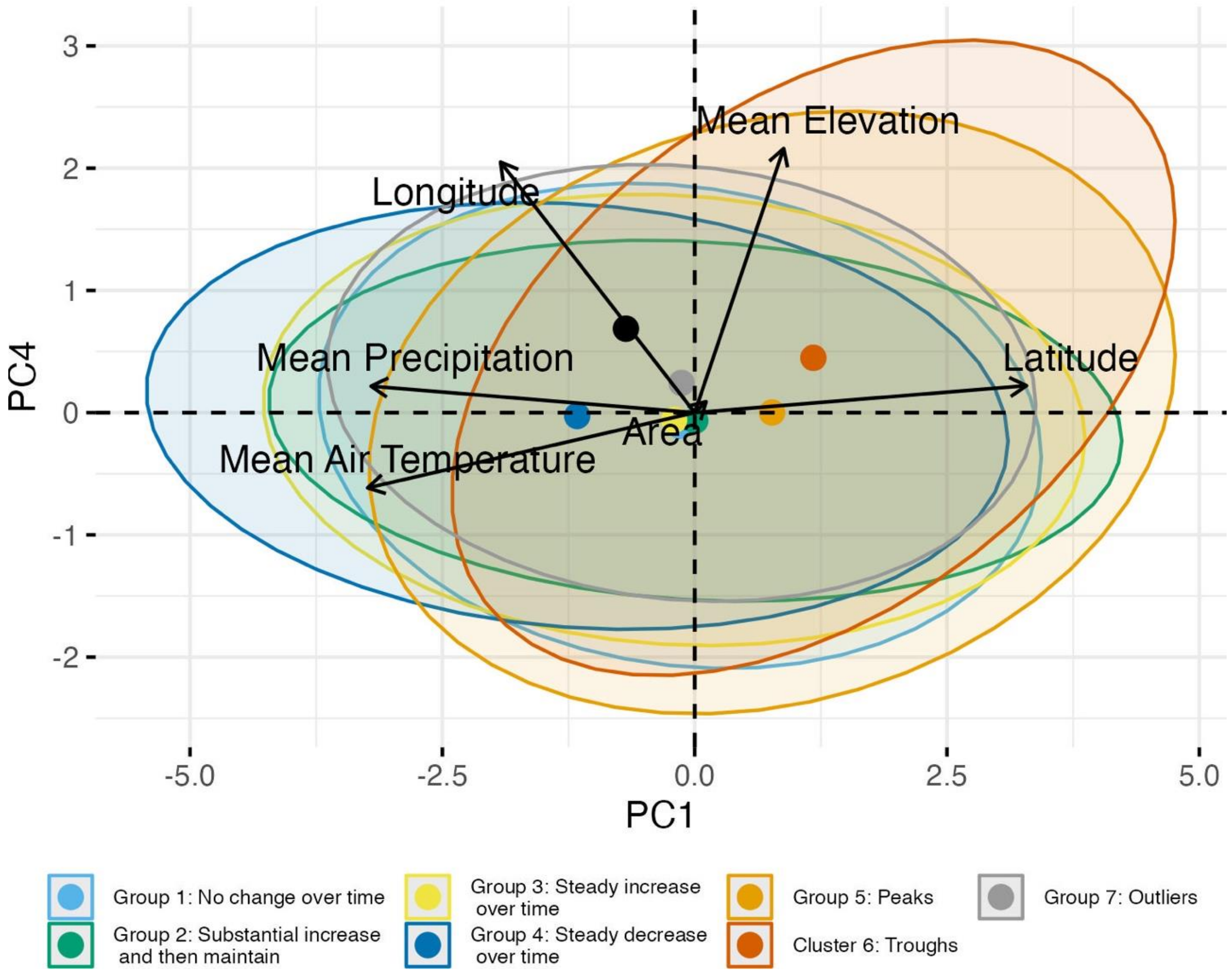

Figure S7. PCA biplot of all seven waterbody groups identified via knowledge-guided machine learning with various environmental drivers. Eigenvectors depict correlations between driver data and each of the seven groups. The colored points represent the centroid of each group and the black point represents the centroid of all 103,930 waterbodies. The principal component on the x-axis (PC1) accounts for 43.8% of the total variance, whereas the principal component on the y-axis (PC4) accounts for 11.4% of the total variance within the dataset.

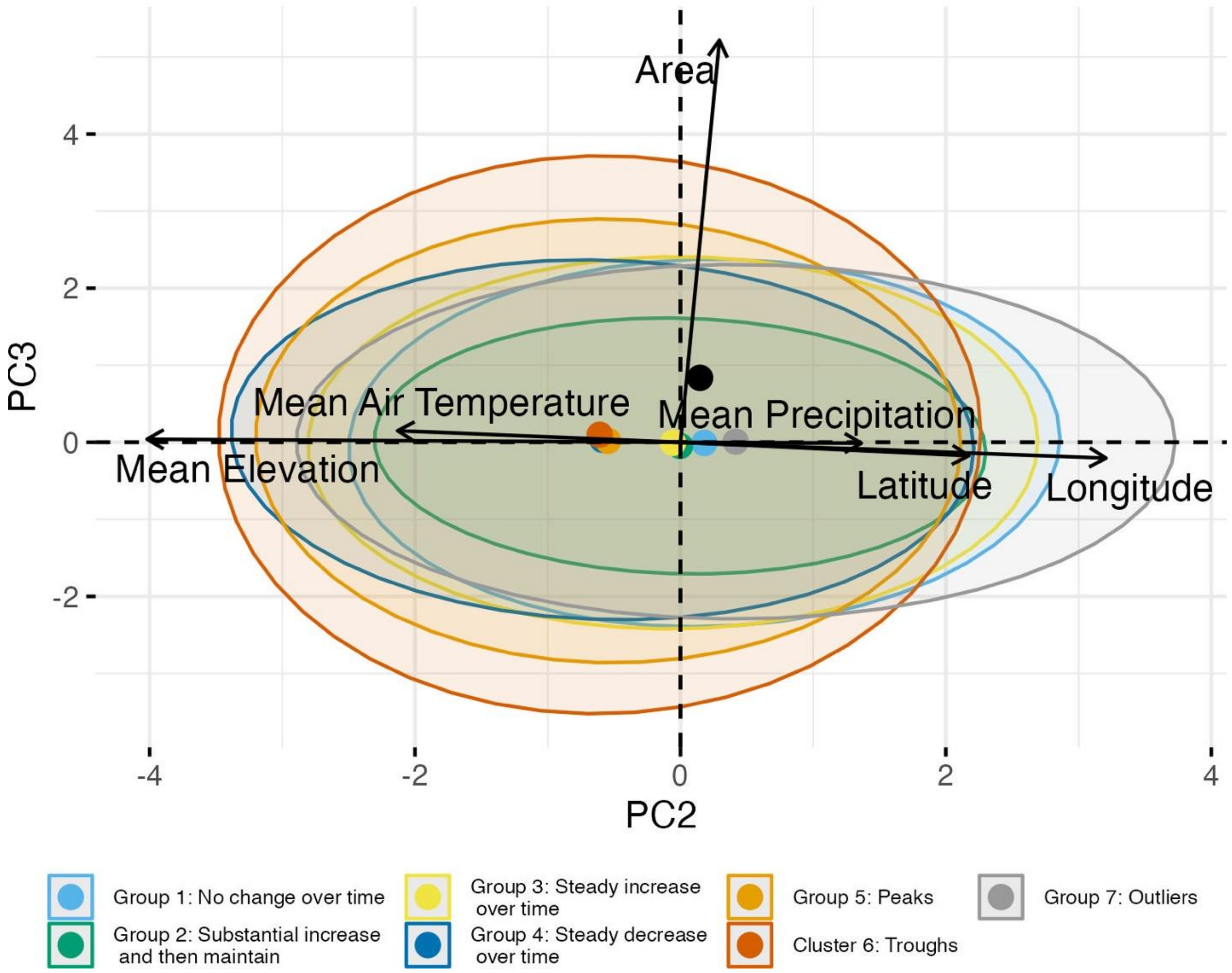

Figure S8. PCA biplot of all seven waterbody groups identified via knowledge-guided machine learning with various environmental drivers. Eigenvectors depict correlations between driver data and each of the seven groups. The colored points represent the centroid of each group and the black point represents the centroid of all 103,930 waterbodies. The principal component on the x-axis (PC2) accounts for 22.9% of the total variance, whereas the principal component on the y-axis (PC3) accounts for 16.6% of the total variance within the dataset

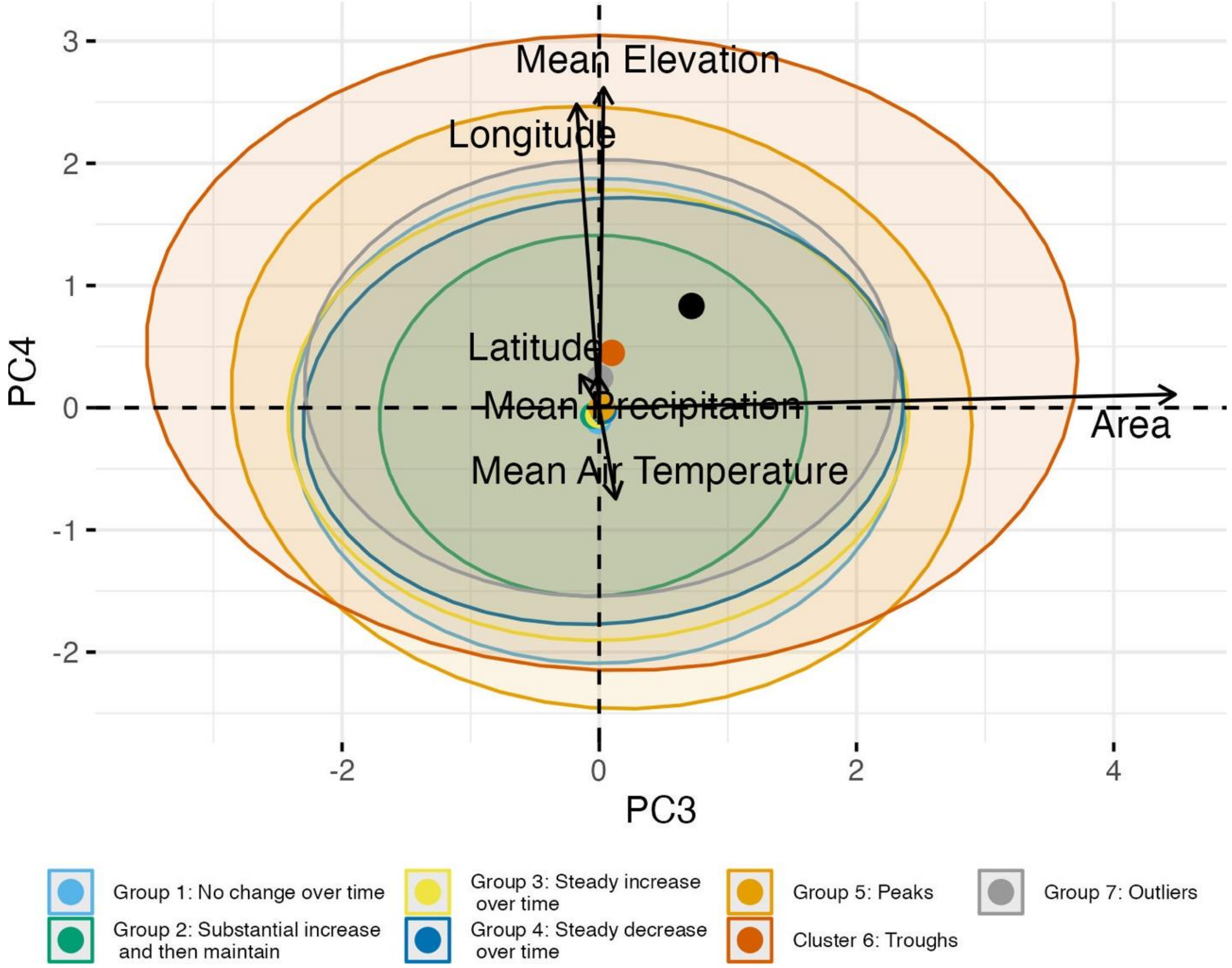

Figure S9. PCA biplot of all seven waterbody groups identified via knowledge-guided machine learning with various environmental drivers. Eigenvectors depict correlations between driver data and each of the seven groups. The colored points represent the centroid of each group and the black point represents the centroid of all 103,930 waterbodies. The principal component on the x-axis (PC3) accounts for 16.6% of the total variance, whereas the principal component on the y-axis (PC4) accounts for 11.4% of the total variance within the dataset.

References:

(1) Ding, C.; He, X. K-Means Clustering via Principal Component Analysis. In *Proceedings of the twenty-first international conference on Machine learning*; **2004**; p 29. https://doi.org/10.1145/1015330.1015408.
(2) Danielson, J. J.; Gesch, D. B. TEMIS -- GMTED2010 Elevation Data at Different Resolutions, **2011**. https://www.usgs.gov/centers/eros/science/usgs-eros-archive-digital-elevation-global-multi-resolution-terrain-elevation. (Accessed Jan 2022).
(3) Willmott, C. J.; Matsuura, K. Terrestrial Air Temperature and Precipitation: 1900-2014 Gridded Monthly Time Series: NOAA Physical Sciences Laboratory Terrestrial Air Temperature and Precipitation: 1900-2014 Gridded Monthly Time Series, **2015**. https://psl.noaa.gov/data/gridded/data.UDel_AirT_Precip.html. (Accessed Jan 2022).
(4) Oksanen, J.; Simpson, G. L.; Blanchet, F. G.; Kindt, R.; Legendre, P.; Minchin, P. R.; O'Hara, R. B.; Solymos, P.; Stevens, M. H. H.; Szoecs, E.; Wagner, H.; Barbour, M.; Bedward, M.; Bolker, B.; Borcard, D.; Carvalho, G.; Chirico, M.; Caceres, M. D.; Durand, S.; Evangelista, H. B. A.; FitzJohn, R.; Friendly, M.; Furneaux, B.; Hannigan, G.; Hill, M. O.; Lahti, L.; McGlinn, D.; Ouellette, M.-H.; Cunha, E. R.; Smith, T.; Stier, A.; Braak, C. J. F. T.; Weedon, J. Vegan: Community Ecology Package, **2022**. https://github.com/vegandevs/vegan.
(5) Anderson, M. J.; Walsh, D. C. I. PERMANOVA, ANOSIM, and the Mantel Test in the Face of Heterogeneous Dispersions: What Null Hypothesis Are You Testing? *Ecol. Monogr.* **2013**, *83* (4), 557–574. https://doi.org/10.1890/12-2010.1.
(6) R Core Team. R: A Language and Environment for Statistical Computing, **2022**. https://www.R-project.org/.
(7) Wander, H. L.; Farruggia, M. J.; La Fuente, R. S.; Korver, M. C.; Chapina, R.; Robinson, J.; Bah, A. R.; Munthali, E.; Ghosh, R.; Stachelek, J.; Khandelwal, A.; Hanson, P. C.; Weathers, K. C. Using Knowledge-Guided Machine Learning to Assess Patterns of Areal Change in Waterbodies across the Contiguous U.S.: Data, **2023**. https://doi.org/10.5281/zenodo.7963296.
(8) Wander, H. L.; Farruggia, M. J.; La Fuente, R. S.; Korver, M. C.; Chapina, R. J.; Robinson, J.; Bah, A. R.; Munthali, E.; Ghosh, R.; Stachelek, J.; Khandelwal, A.; Hanson, P. C.; Weathers, K. C. Using Knowledge-Guided Machine Learning to Assess Patterns of Areal Change in Waterbodies across the Contiguous US, **2023**. https://zenodo.org/records/7986852.
(9) Dingman, S. L. Elevation: A Major Influence on the Hydrology of New Hampshire and Vermont, USA / L'altitude Exerce Une Influence Importante Sur l'hydrologie Du New Hampshire et Du Vermont, Etats-Unis. *Hydrol. Sci. Bull.* **1981**, *26* (4), 399–413. https://doi.org/10.1080/02626668109490904.
(10) Pepin, N.; Bradley, R. S.; Diaz, H. F.; Baraer, M.; Caceres, E. B.; Forsythe, N.; Fowler, H.; Greenwood, G.; Hashmi, M. Z.; Liu, X. D. Elevation-Dependent Warming in Mountain Regions of the World. Nat Clim Chang 5: 424–430, **2015**.
(11) Firatli, E.; Dervisoglu, A.; Yagmur, N.; Musaoglu, N.; Tanik, A. Spatio-Temporal Assessment of Natural Lakes in Turkey. *Earth Sci. Inform.* **2022**, *15* (2), 951–964. https://doi.org/10.1007/s12145-022-00778-8.
